# On the Liquid Chemistry of the Reactive Nitrogen Species Peroxynitrite and Nitrogen Dioxide Generated by Physical Plasmas

**DOI:** 10.3390/biom10121687

**Published:** 2020-12-16

**Authors:** Giuliana Bruno, Sebastian Wenske, Jan-Wilm Lackmann, Michael Lalk, Thomas von Woedtke, Kristian Wende

**Affiliations:** 1 Centre for Innovation Competence (ZIK) Plasmatis, Leibniz Institute for Plasma Science and Technology (INP Greifswald), 17489 Greifswald, Germany; giuliana.bruno@inp-greifswald.de (G.B.); sebastian.wenske@inp-greifswald.de (S.W.); 2 Cluster of Excellence Cellular Stress Responses in Aging-Associated Diseases, University of Cologne, 50931 Cologne, Germany; jan-wilm.lackmann@uni-koeln.de; 3 Institute of Biochemistry, University of Greifswald, 17487 Greifswald, Germany; lalk@uni-greifswald.de; 4 Leibniz Institute for Plasma Science and Technology, 17489 Greifswald, Germany; woedtke@inp-greifswald.de

**Keywords:** cold physical plasmas, kINPen, redox signaling, reactive nitrogen species, nitrosative stress, oxidative post-translational modifications

## Abstract

Cold physical plasmas modulate cellular redox signaling processes, leading to the evolution of a number of clinical applications in recent years. They are a source of small reactive species, including reactive nitrogen species (RNS). Wound healing is a major application and, as its physiology involves RNS signaling, a correlation between clinical effectiveness and the activity of plasma-derived RNS seems evident. To investigate the type and reactivity of plasma-derived RNS in aqueous systems, a model with tyrosine as a tracer was utilized. By high-resolution mass spectrometry, 26 different tyrosine derivatives including the physiologic nitrotyrosine were identified. The product pattern was distinctive in terms of plasma parameters, especially gas phase composition. By scavenger experiments and isotopic labelling, gaseous nitric dioxide radicals and liquid phase peroxynitrite ions were determined as dominant RNS. The presence of water molecules in the active plasma favored the generation of peroxynitrite. A pilot study, identifying RNS driven post-translational modifications of proteins in healing human wounds after the treatment with cold plasma (kINPen), demonstrated the presence of in vitro determined chemical pathways. The plasma-driven nitration and nitrosylation of tyrosine allows the conclusion that covalent modification of biomolecules by RNS contributes to the clinically observed impact of cold plasmas.

## 1. Introduction

Reactive nitrogen and oxygen species (RNS/ROS) are unstable compounds prone to react rapidly with cellular molecules. In biological systems, they may be involved in redox signaling pathways (e.g., oxygen sensing, muscles and vascular physiology, immune defense, inflammatory processes) [1], mostly by covalently changing the structure of biomolecules such as lipids and proteins [2,3,4,5]. When their homeostasis is impaired, they become markers or drivers of pathological conditions like cancer progression as well as metabolic and neurodegenerative diseases. While some species are constantly formed as by-products of cell metabolism, others are generated for dedicated purposes (second messengers, oxidative burst) [4,6,7,8,9]. Well-known endogenous radicals are hydroxyl radicals (˙OH); superoxide anion (˙O_2_^−^); nitric oxygen radicals (˙NO); nitric dioxide radicals (˙NO_2_); and other reactive species like singlet oxygen (^1^O_2_), hydrogen peroxide (H_2_O_2_), peroxynitrite (ONOO^−^), dinitrogen trioxide (N_2_O_3_), and the nitrite ion (NO_2_^−^). The dual role of ROS and RNS is exploited by various strategies aiming to control or modulate redox-signaling pathways [10,11,12,13]. Among these, cold plasma discharges, ionized gases with complex physical and chemistry properties, are being investigated [14,15]. Most prominent components are small reactive species, many of which are also endogenously generated. A large number of gas plasma sources with different gas phase chemistry have been developed [16,17]. One of the best-characterized plasma sources is the kINPen, approved as certified medical tool in the medical version (kINPen MED) [18], currently applied in research (cancer immunology) and in clinics (wound care, skin-related diseases) [19,20,21,22,23,24]. To the current knowledge, by tuning central parameters of the discharge including working gas composition, distance, or power, the variation of the resulting plasma chemistry is responsible for the observed biological and biochemical effects [25,26,27,28,29]. In addition to direct signaling events triggered by long-lived reactive species like H_2_O_2_, e.g., via peroxiredoxins, plasma-derived short-lived reactive species can covalently modify biomolecules in model systems [26,30,31,32,33,34,35]. It remains to be clarified whether extracellularly modified molecules yield to intracellular physiological consequences or if only changes in cellular structures (e.g., cell membrane proteins) are relevant.

Gas plasmas can be tuned to create high levels of oxygen species, such as singlet oxygen ^1^O_2_; atomic oxygen ˙O; ozone O_3_; and, to a lesser extent, hydrogen peroxide H_2_O_2_ and hydroxyl radicals ˙OH. These conditions are applied for disinfection or cancer regression [36,37,38,39,40]. In contrast, other conditions foster an N_2_-driven chemistry, yielding species like ˙NO, ˙NO_2_, N_3_O_5_, HNO_2_, and HNO_3_ [41,42]. The nitrogen species are assumed to play a role in bactericidal [43,44] and virucidal effects [45], as well as cell stimulation, e.g., in wound healing [11,46,47,48,49,50,51,52]. In contrast, data on their transport, solubilization, or production in liquids, as well as their impact on biological systems, are limited. While the long-lived species nitrite and nitrate can be easily accessed by the Griess assay or the ion chromatography and are almost inevitably observed for plasma discharges, both ions are in physiologic conditions not reactive enough to contribute significantly to the effects seen after plasma treatments. Nitration [28,53,54] and nitrosylation [35] of biomolecules have been observed, indicating the presence (and activity) of more reactive species at least under certain conditions. Potential candidates are, e.g., ˙NO_2_, N_2_O_3_, and ONOO^−^. The presence of peroxynitrite [43,54] and peroxynitrate [55] has been reported for liquid systems under acidic conditions (pH < 4.5), as well as in alkaline conditions (pH 12) in the case of peroxynitrite [56], while information on neutral environments is missing. However, using an peroxynitrite sensitive europium probe, intracellular peroxynitrite was recently detected in human cell lines following a plasma treatment [57]. Natural sources of RNS are enzymes of the nitric oxide synthase (NOS) complex, generating ˙NO. This RNS can react with O_2_ yielding oxidized RNS such as ˙NO_2_ and N_3_O_5_, or with superoxide anion radicals produced by mitochondria, NADPH-oxidases, or xanthine oxidase, forming peroxynitrite [58]. Because of the capability of reactive nitrogen species to modify biological structures and to modulate signaling pathway, e.g., via acting as a second messenger like nitric oxide, a significant contribution of RNS can be expected [59].

Tyrosine is a key amino acid reactive towards RNS and protein tyrosines are significant targets in biological environments [60,61,62]. The principal roles of tyrosine are the formation of hydrophobic cores in proteins, as well as the transduction of signals via phosphorylation events. In this, tyrosine has a superior relevance compared with serine or threonine because of the high specificity of some protein kinases [63]. The presence of the aromatic hydroxyl group also allows the reaction with non-protein structures, in contrast to phenylalanine. Therefore, modifications of the tyrosine substructure yield a gain or loss of function of proteins and subsequently alter signaling pathways or immunological responses [61,64]. Protein nitration also acts as a signal for negative feedback regulation, leading to ubiquitination and protein degradation [65,66]. Some studies suggest the involvement of tyrosine nitration in the pathogenesis or as marker of nitro-oxidative stress [65,67,68,69]. Nitric oxide and its oxidation products are also involved in wound healing processes, although the mechanisms are still under investigation [70,71]. For example, tyrosine nitration occurring on metalloproteinase-13 (MMP-13) promoted its release by endothelial cells, triggering a faster cell migration, angiogenesis, and wound healing [67]. In the same way, it was shown that protein tyrosine nitration by exogenous ˙NO donors inhibited the degranulation processes in mast cell lines [72]. The release of exogenous ˙NO is a possible strategy to stimulate skin healing [73], potentially beneficial in diabetic patients, where the increased hyperglycemia leads to decreased ˙NO bioavailability and delays the wound healing process [74]. The effectiveness of cold plasmas was shown in the treatment of wounds in both diabetic and normal subjects, as well as in wound models [22,23,75,76]. The responsible species are not completely known, but recent observations emphasized the role of the local cell stimulation over wound bed disinfection—suggesting that reactive nitrogen species may be involved. Therefore, this work intends to further investigate the N_2_-driven biochemistry induced by the kINPen plasma source, aiming to define the principal formed bioactive reactive species and their mechanisms of action. To reach this aim, tyrosine was chosen as a tracer molecule and modifications occurring as a result of the impact of plasma-derived reactive nitrogen species were identified and quantified via high-resolution mass spectrometry. Finally, the ability of kINPen plasmas in modulating the RNS-mediated pathways in complex models was tested. Overall, a significant impact of nitrosating and nitrosylating agents, assumingly peroxynitrite, was observed, leading to a covalent chemical modification of sensitive target structures. Given this result, functional consequences will arise in both pro- and eukaryotic cells, highlighting a possible key mechanism in promoting wound healing, modulation of cancer immunology, and antimicrobial effects.

## 2. Materials and Methods

### 2.1. Sample Preparation

#### 2.1.1. Model Solutions

L-tyrosine (Merck KGaA, Darmstadt, Germany) solutions with a concentration of 0.3 and 30 mM were prepared in 5 mM ammonium formate buffer pH 7.4 (Merck KGaA, Darmstadt, Germany) fresh and kept on ice until use. The singlet oxygen and superoxide scavenger ergothioneine (Enzo Life Sciences GmbH, Lörrach, Germany) and the nitric oxide scavenger cPTIO (Lot. PK822, Dojindo Molecular Technologies, Kumamoto, Japan) were dissolved in double distilled water to create a 10× stock solution with 3 mM concentration [77,78,79].

#### 2.1.2. Wound Exudates

Wound exudates were obtained from seven diabetic patients with chronic wounds of the Klinikum Kalsburg, Germany according to an existent ethics approval. After removal of the wound bandage material, the patient wound was rinsed with sterile natural saline followed by blotting with a dry gauze. Afterwards, Copan eSwab samples (Mast Diagnostica GmbH, Reinfeld, Germany) were taken in a circular, inside-out motion across the treated wound surface, and rotated during sampling. Samples were placed in 1000 µL of serum-free Roswell Park Memorial Institute (RPMI) 1640 medium (Thermo Scientific, Rockford, IL, USA). Subsequently, wounds were plasma treated (see Section 2.2), rinsed again, and sampled as before using eSwabs. The swabs were vortexed for the maximal elution of wound exudates into the medium, and the supernatant was centrifuged at 4500 rpm for 5 min and sterile-filtered (0.22 µm). All swabs and supernatants were kept at −80 °C until further use.

### 2.2. Cold Plasma Treatments and Incubation with Control Oxidants

**Figure 1:**
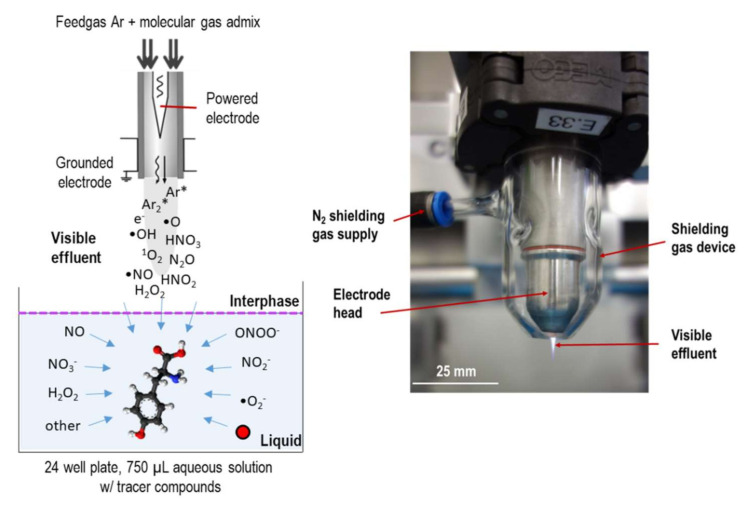
Schematic layout of treatment of tyrosine model solutions (**left**), and kINPen device w/gas shield installed (**right**).

For the model solutions, the kINPen09 (neoplas tools GmbH, Greifswald, Germany) with a shielding device was used as a source of reactive species, as shown in Figure 1. Briefly, 750 µL of tyrosine solutions was treated in 24-well plates at a distance of 9 mm for 30 or 180 seconds. Argon (±1% of molecular gas admixture, Air Liquide, Paris, France) served as working gas using 3 standard liters per minute (slm). Admixtures were 1% oxygen, 1% nitrogen, or a mix of both (0.7% N_2_ and 0.3% O_2_, Air Liquide, Paris, France). For some treatments, isotopically labelled working gas admixtures (^15^N_2_, or ^18^O_2,_ Merck KGaA, Darmstadt, Germany) or solvent (water, H_2_^18^O, Merck KGaA, Darmstadt, Germany) were used. The plasma effluent was shielded against the ambient air by a gas shielding of 5 slm nitrogen. For some treatments, the working gas was enriched with water (320 ppm) by guiding 1% of the total flow through a gas wash bottle containing double distilled water [41,80,81]. Chronic wounds were treated in the Klinikum Karlsburg using the kINPen MED (neoplas tools GmbH, Greifswald, Germany) under standard medical conditions (30 s per 1 cm^2^ of wound area and 5 slm of Argon (Air Liquide, Paris, France), without a shielding device.

**Figure 2:**
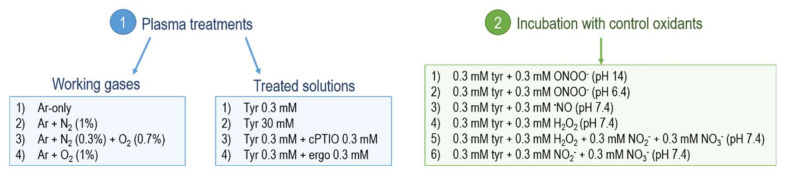
Overview of the solutions treated with plasmas (1) and incubated with control oxidants (2). Here, 3 slm of every working gas applied, together with 5 slm N_2_ shielding gas. Prepared solutions were buffered with 5 mM ammonium formiate. Tyrosine (Tyr); ergothioneine (ergo).

Buffered solutions (pH 7.4) of tyrosine 0.3 mM were incubated for 2 min at room temperature under constant mixing with equimolar amounts of control oxidants (300 µM). Those were (1) peroxynitrite (Merck KGaA, Darmstadt, Germany) at pH 14; (2) peroxynitrite at pH 6.4, yielding 30% peroxynitrous acid dissociation in nitric dioxide radicals and hydroxyl radicals [54,82,83]; (3) the nitric oxide donor DEA NONOate (Biomol GmbH, Hamburg, Germany); (4) nitrite and nitrate (Merck KGaA, Darmstadt, Germany); (5) hydrogen peroxide (Merck KGaA, Darmstadt, Germany); and (6) mixed solutions of nitrite, nitrate, and hydrogen peroxide. For solutions (5) and (6), 300 µM of each control oxidant was included. After reaction, samples were put on ice and immediately subjected to mass spectrometry analysis. An overview of all the plasma treatment conditions (working gases and treated solutions) and of the solutions incubated with control oxidants is shown in Figure 2.

### 2.3. Cold Plasma-Induced Modifications of Tyrosine

#### 2.3.1. Qualitative Screening via Direct Infusion High-Resolution Mass Spectrometry

All samples were analyzed by high-resolution mass spectrometry using a TripleTOF 5600 (AB Sciex GmbH, Darmstadt, Germany). Samples were diluted 1:1 with acetonitrile/0.1% formic acid to stimulate ionization and to facilitate evaporation and injected using a syringe pump (50 µL/min). Ionization was achieved by positive mode electro spray ionization with the following settings: +5.5 kV probe voltage, 300 °C, +30 V declustering potential, 40 psi curtain gas, 20 psi gas 1, and 25 psi gas 2 (Turbo Ion Source). Concentrated sample solutions (30 mM) were diluted 1:100 before injection. All spectra were acquired in the m/z range from 40 to 600 m/z. After a first qualitative scan of the produced compounds, their structures were elucidated by acquisition in tandem mass spectrometry (MS^2^ spectra) and collisional energies for optimal fragmentation were tuned for each compound of interest.

#### 2.3.2. HPLC-MS^2^ Quantitation of Tyrosine and 3-Nitrotyrosine

Considering their relevance, L-tyrosine and 3-nitro-L-tyrosine (Merck KGaA, Darmstadt, Germany) were absolutely quantified by coupling a chromatographic separation (Infinity II 1290, Agilent Technologies, Berlin, Germany) to the mass spectrometry detection (qTRAP 5500, AB Sciex GmbH, Darmstadt, Germany). A hydrophilic liquid chromatography (HILIC) strategy was adopted, using a 2.1 mm × 5 mm Acquity Amide VanGuard Pre-column followed by a 2.1 mm × 100 mm Acquity Amide Column (both 130 Å pore size, 1.7 µm particle size, Waters Corporation, Berlin, Germany). For the separation, eluent A (85% acetonitrile, 0.15% of formic acid, and 10 mM ammonium formate) and eluent B (HPLC water, 0.15% formic acid, and 10 mM ammonium formate; pH 3), were used. A linear gradient at a flow rate of 800 µL/min was applied (time, B): 0 min, 99%; 4 min, 92%; 4.1 min, 99%; 5 min, 99%. After a 1:5 dilution in buffer A, 20 µL of sample was injected. Ionization was achieved in positive mode, using the following parameters: +5.5 kV probe voltage at 150 °C, +100 V declustering potential, 35 psi curtain gas, 25 psi gas 1, and 30 psi gas 2. Compounds of interest were quantified by multiple reaction monitoring. The transitions and collisional energies (CEs) were tuned differently for each compound: tyrosine 182 → 136 m/z (quantitative), CE 18 V and 165 m/z (qualitative), CE 10 V. Nitro-tyrosine 227 → 181 m/z (quantitative) and 158 m/z (qualitative), both CE 10 V. For the quantification, external calibration curves were generated.

#### 2.3.3. Data Analysis and Visualization

All experiments were performed three times each with technical duplicates. Statistical analysis was performed using GraphPad Prism 7. The MarvinSketch software (version 18.8.0) was used to identify the exact molecular weight and the formula of each compound, as well as to predict the reactivity of tyrosine and control oxidants in different pH. The spectra produced through direct infusion high-resolution mass spectrometry were calibrated and analyzed with PeakView (version 1.2.0.3, AB Sciex GmbH, Darmstadt, Germany). The peak areas of each observed derivative were normalized on the peak area of the control (untreated tyrosine), giving a relative estimate of the conversion. For treatments involving isotopes, the measurement data were corrected according to [36], taking into account the natural distribution of isotopes and the purity grade.

### 2.4. Characterization of Plasma-Induced Protein Modifications in Wound Exudates

Proteins were precipitated by incubating with 80% ice-cold acetone (Carl Roth GmbH, Karlsruhe, Germany) overnight. The pellets were washed twice and dissolved in 100 µL resuspension buffer (10 mM Tris/HCl with 1 mM EDTA, pH 8, all Merck KGaA, Darmstadt, Germany). Protein concentrations were determined by Bradford assay following the vendor’s high sensitivity protocol (Roti-Nanoquant, Roth, Germany). Single dimension electrophoresis was achieved by loading 60 µg of each sample onto 10% precast protein gels (Bio-Rad Laboratories, Hercules, CA, USA), followed by in-gel digestion [30]. Briefly, gel slices were dried and incubated for 5 min with 50 µL of 10 mM tris(2-carboxyethyl)phosphine (Merck KGaA, Darmstadt, Germany) at 60 °C followed by an incubation with 50 µL of 50 mM iodoacetamide to alkylate reduced thiols at room temperature (RT) for 30 min (Merck KGaA, Darmstadt, Germany). After brief drying, 2.5 µg trypsin (sequencing grade, Promega GmbH, Mannheim, Germany) was added and samples were incubated at 37 °C for 16 h. Peptides were eluted into ultra-pure water by ultra-sonication for 30 min. The peptides were further purified using Pierce C18 tips (Thermo Fisher Scientific, Hennigsdorf, Germany) following the included protocol for peptide purification and desalting. Eluted peptides were subjected to nanoLC/HRMS. An UltiMate 3000 nanoLC (Dionex Corp., Sunnyvale, CA, USA) was coupled to a QExactive mass spectrometer using electrospray ionisation (both Thermo Fisher Scientific, Hennigsdorf, Germany). Sample aliquots of 1 µg were loaded onto an Acclaim PepMap 100 precolumn (2 cm × 100 µm, 5 µm particle size, 100 Å pore size) for 6 min at 5 µL/min flow followed by separation on a PepMap RSLC column (25 cm × 75 µm, 2 µm particle size, 100 Å pore size). The following gradient was used at 200 nl/min: 2% to 35% in 6 min, to 50% B in 15 min, to 90% B in 15 min, keeping at 90%for 15 min, and equilibration at 2% B for 20 min (A: H_2_O + 0.1% acetic acid, B: acetonitrile + 0.1% acetic acid, both Merck KGaA, Darmstadt, Germany). Each sample was injected twice. The QExactive was run in Top10 DDA mode with a dynamic exclusion of 30 s. MS1 spectra were acquired with a resolution of 70,000, whereas MS2 spectra were acquired in 17,500 resolution. Raw data files were analyzed with the Proteome Discoverer 2.2 (Thermo Fisher Scientific, Hennigsdorf, Germany) software. At least two unique peptides had to be identified with a maximal mass divergence of 5 ppm (MS1) and 0.02 Da (MS2) for the corresponding protein to be accepted. A maximum false discovery rate (FDR) of 5% was accepted for the datasets. Afterwards, abundances were normalized on individual trypsin intensities and resulting relative intensities taken for label free quantification using two-fold cut offs. In a second step, samples were analyzed using the Byonic (Protein Metrics Version 3.6) plug-in for Proteome Discoverer. Here, a list of oxidative chemical modifications was identified using a machine-learning algorithm with a database, which was previously acquired using model peptides [84]. For normalization, the peptide spectrum matches with a modification were counted and divided by the total number of peptide spectrum matches in each of the samples. The modifications found with Byonic were filtered and scored to separate nonsense peptide spectrum matches from those correctly identified.

## 3. Results and Discussion

### 3.1. Tyrosine Modification Induced by Plasma-Generated Reactive Oxygen and Nitrogen Species

Tyrosine solutions were treated by cold plasma with varying parameters or incubated with control oxidants. The generated products were identified via the accurate monoisotopic mass and on the tandem-MS level identifying molecule substructures. All the identified structures are listed in Table 1, independently from the condition in which they were produced. According to [33,34], where tyrosine solutions have been treated with plasma sources operated with air or helium as ionized gases, structures 2, 3, 10, and 22 were also identified in the current work using kINPen plasmas. Besides, more than 20 other types of functionalization were observed. The dominant modifications observed were all localized on the aromatic ring: hydroxylations, nitrosylations, nitrations, and a combination of different groups (up to four groups). Here, the addition of a functional group via electrophilic substitutions is stabilized by resonance effects [85,86]. To a lesser extent, the dimerization of tyrosine to dityrosine and the functionalization of this structure with other groups were also detected. While hydroxylations were mostly driven by oxygen species, the presence of N-containing functional groups indicated an active RNS chemistry. This was partially shown previously using cysteine as tracer, yielding S-nitroso-cysteine [30].

**Figure 3:**
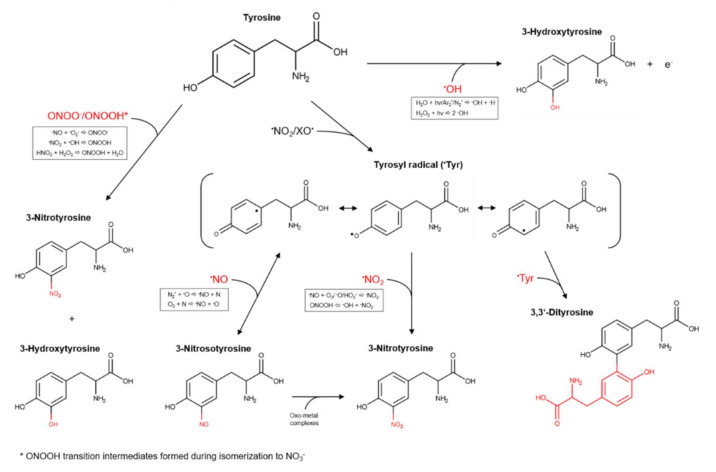
Formation pathways of tyrosine derivatives considering a neutral pH. The hypothesized possible responsible species generated by kINPen plasmas and the reactions leading to their origin are highlighted in the box [15,65,81,88,89].

However, the detected amounts were low, suggesting that cysteine is not an optimal target for the intended downstream analysis technique. The number and amount of identified N-containing modifications of tyrosine show a good suitability to study the RNS output of cold plasma discharges. An overview of oxidative modifications induced on tyrosine by different reactive species, possibly also formed by kINPen plasmas, is shown in Figure 3. When considering a radical-driven reaction mechanism, the first step for tyrosine derivatization is the formation of tyrosyl radicals by different one-electron oxidants (˙NO_2_, ˙OH). ˙NO_2_ is a candidate species able to form tyrosyl radicals via a slow reaction (k = 3.2 × 10^5^ M^−1^ s^−1^), while hydroxyl radicals react at rates ≥1 × 10^9^ M^−1^ s^−1^. Consequential further direct reactions of tyrosyl radicals with tyrosine yield dityrosine (k = 2.3 × 10^8^ M^−1^ s^−1^); those with ˙NO yield nitrosotyrosine (k = 1.0 × 10^9^ M^−1^ s^−1^); those with ˙NO_2_ yield nitrotyrosine (k = 3.0 × 10^9^ M^−1^ s^−1^); and those with ˙O_2_^−^ yield tyrosine hydroxyquinone (k = 1.5 × 10^9^ M^−1^ s^−1^) (only on free tyrosines). This last product rapidly loses O_2_ from the structure to reform tyrosine [59,65,87]. After the formation of those derivatives, a further addition of groups led by reactive species was assumed. The conversion of NO-tyrosine to NO_2_-tyrosine can occur in oxidative conditions firstly by formation of an iminoxyl radical (˙NO-tyrosine) and, further, oxygen addition. This two one-electron oxidation step process is a slow one, promoted by the presence of metals, which are not included in the used liquid model. In contrast, a direct reaction with ˙OH (and possibly ˙O) leads to the formation of tyrosine hydroxyl radicals (˙OH-tyrosine) (k = 1.2 × 10^10^ M^−1^ s^−1^), which rapidly lose an electron to become OH-tyrosine [65].

A non-radical mechanism driven by peroxynitrous acid and peroxynitrite has also been proposed by [88], leading to tyrosine hydroxylation and nitration by formation of ONOOH transition intermediates during the H^+^ driven isomerization of peroxynitrous acid to NO_3_^−^ and H^+^. Indeed, the energy and the rate for the isomerization (18 kcal mol^−1^ and 1.3 s^−1^, respectively) are equivalent to those necessary to achieve the nitration or hydroxylation of aromatic compounds, occurring independently from their concentrations. At very low pH (<2.5), the formation of nitryl anion (NO_2_^+^) by peroxynitrous acid or peroxynitrite heterolysis could occur and lead to tyrosine nitration [89]. However, this mechanism can be excluded in our system, because of the controlled pH at 7.4.

### 3.2. Gas Composition: A Crucial Parameter to Regulate the NO_x_ Generation

**Figure 4:**
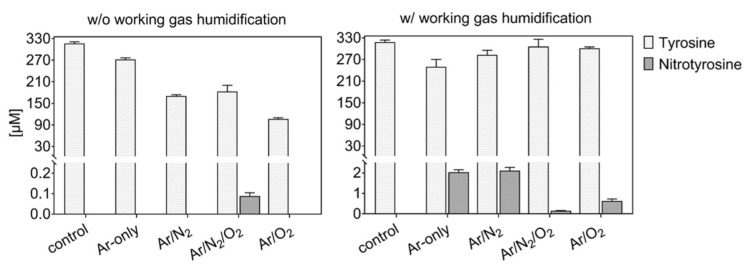
Tyrosine consumption and 3-nitrotyrosine formation by plasma-derived reactive oxygen species (ROS). The presence of water in the working gas reduced gas phase oxidation of reactive nitrogen species (RNS) and yielded higher activity of RNS in the liquid (3 min, 0.3 mM tyrosine in 5 mM ammonium formate, pH 7.4). Plasma is formed from dry (left) or humid (320 ppm H_2_O, right) working gas.

**Figure 5:**
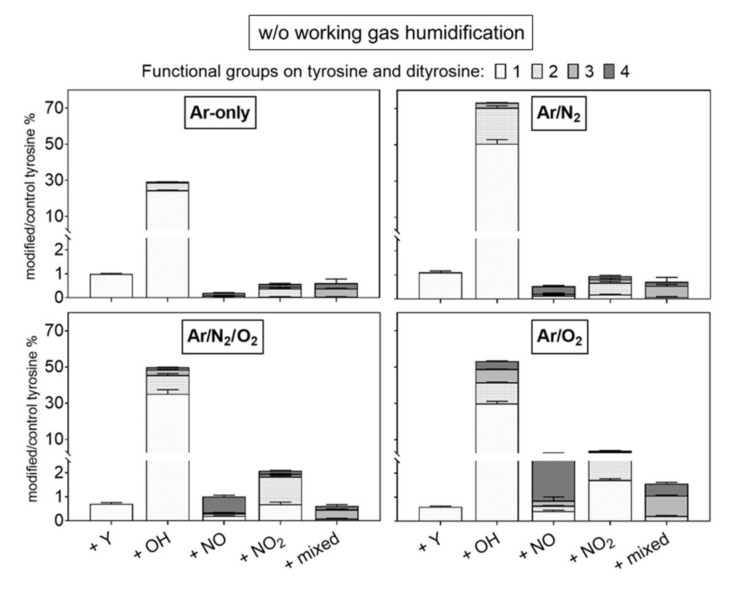
Modifications of tyrosine observed after plasma treatment using dry working gas are dominated by hydroxylation. Multiple nitrations/nitrosylations were observed predominantly when O_2_ was present in the working gas. Labels: +Y (tyrosyl, +180.066 Da), +OH (hydroxyl, +15.9949 Da), +NO (nitroso, +28.9902 Da), and +NO_2_ (nitro, +44.9851 Da). Up to four groups were observed per molecule (indicated by light to dark grey). The introduction of diverse groups is represented as “mixed” (Compounds 20 to 27, Table 1). Treatment time 3 min, 0.3 mM tyrosine in 5 mM ammonium formate, pH 7.4. Relative compound intensities are given (tyrosine ~4445 counts in control).

**Figure 6:**
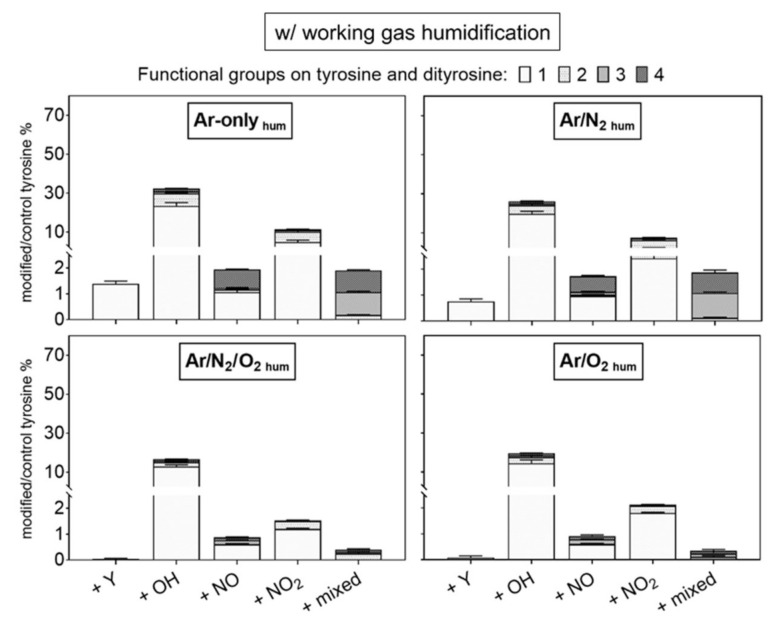
Modifications of tyrosine observed after plasma treatment using humidified working gas show a substantial introduction of one nitroso or nitro group. Labels: +Y (tyrosyl, +180.066 Da), +OH (hydroxyl, +15.9949 Da), +NO (nitroso, +28.9902 Da), and +NO_2_ (nitro, +44.9851 Da). Up to four groups were observed per molecule (indicated by light to dark grey). The introduction of diverse groups is represented as “mixed” (Compounds 20 to 27, Table 1). Treatment time 3 min, 0.3 mM tyrosine in 5 mM ammonium formate, pH 7.4. Relative compound intensities are given (tyrosine ~4445 counts in control).

Tyrosine solutions were treated using different working gas compositions and treatment times, yielding various products. While tyrosine and 3-nitrotyrosine were absolutely quantified by a multiple reaction monitoring approach (Figure 4), all other tyrosine derivatives were relatively quantified (Figure 5—dry working gas, Figure 6—humidified working gas).

The highest tyrosine consumption (68%) was observed for a discharge regime rich in short-lived ROS (dry Ar/O_2_) [25]. In contrast, hydrogen peroxide rich conditions (dry Ar) [81] yielded only 14% tyrosine conversion.

Using admixtures of molecular gases, more than 50% of tyrosine was converted into hydroxytyrosine. Target water ionization, homolysis, or photolysis is promoted by excited states of Ar_2_ or N_2_ (excimers), as well as from radical reactions [36,90]. The direct reaction with those species (or for excimers, with their radiation) leads to the predominant formation in water of ˙OH and ˙H [91,92,93], which would react directly with tyrosine to form hydroxytyrosine. The higher OH-tyrosine production in conditions with Ar/N_2_ (up to 70% converted tyrosine) confirmed the synergistic action on the target of Ar_2_ and N_2_ excimers [15]. In contrast, in conditions including oxygen, gaseous radicals (e.g., ˙O) are predominantly formed, and could be responsible for tyrosine hydroxylation (up to 4 −OH groups) via direct reaction with tyrosine or via water dissociation and ˙OH formation in liquid [94,95]. In the presence of both N_2_ and O_2_ in the working gas, a substantial consumption of tyrosine was observed (≈5%), alongside the detection of only small amounts of 3-nitrotyrosine in dry conditions (Figure 4).

Accordingly, the formation of primary or secondary nitrosative NO_x_ species can be assumed for this condition, corroborating previous results determining S-nitrosocysteine formation. Here, the presence of ˙NO in the liquid did not yield in *S*-nitrosylation, and ˙NO oxidation products (e.g., ONOO^−^, N_2_O_3_) were assumed to be of relevance. Considering the strong accumulation of nitrate in this discharge condition ], an end product that can derive from the decomposition of peroxynitrite, ONOO^−^ could be responsible for both S-nitrosylation and nitrotyrosine formation [96]. The incorporation of two −NO_2_ (%) and four −NO (up to 5%) groups was detected in major amounts in admixtures including oxygen (Figure 5), confirming an active nitrogen chemistry in the liquid also using shielding N_2_ as a source [42,97]. Considering quantitative data, an isobaric structure for nitrotyrosine (e.g., hydroxy-nitrosotyrosine, see Table 1) was detected in conditions with oxygen only as admix. Previous simulation studies showed the highest gas phase formation of ˙NO_2_ (~8 × 10^13^ cm^−3^) and N_2_O (~5 × 10^12^ cm^−3^) using dry working gases (N_2_ shielded) containing 1 % N_2_/O_2_ with O_2_ in less than 50%. Those species decreased by increasing the O_2_ %, with the increase of highly oxidized species, such as O_3_ (~1.5 × 10^15^ cm^−3^) and N_2_O_5_ (~3 × 10^13^ cm^−3^) [81]. Considering the higher production of nitrotyrosine in conditions with 1:1 N_2_/O_2_ admixtures (Figure 4), ˙NO_2_ could be a direct nitrating agent of tyrosyl radicals in the target. However, it must be considered that only a minimal amount of ˙NO_2_ would be able to diffuse from the gas phase into the bulk of the liquid, owing to its low solubility in water (H^cp^ = 3.4 × 10^−2^ Pa^−1^) [98]. Most likely, a direct nitration due to gaseous ˙NO_2_ could occur at the interface. These limitations could justify the overall low production of nitrotyrosine.

Alternatively, the production of other nitrating agents that have ˙NO_2_ as precursor can be considered. The reaction between ˙NO_2_ and water molecules generates HNO_2_, which is highly soluble [35]. However, at a pH of 7.4, the nitrous acid cannot be considered as a nitrating agent. ˙NO_2_ could form N_2_O_3_, a nitrating agent, by reaction with ˙NO, which is present in gas and liquid phase [81]. However, because of the low solubility of N_2_O_3_ (H^cp^ = 6.0 × 10^−1^ Pa^−1^) [98], its penetration to the target is unlikely. With that, the well soluble peroxynitrite could be a prominent candidate for the effective nitrating species, acting on tyrosine via dissociation in ˙NO_2_ [65], or transition intermediates of peroxynitrous acid formed during its isomerization in nitrate [99]. ONOOH/ONOO^−^ production is promoted by reaction of gaseous ˙NO and ˙O_2_^−^ [81], but considering ˙NO_2_ as major gaseous precursor, interface/bulk reactions of ˙NO_2_ (gas) with ˙OH (in liquid) or HNO_2_ with H_2_O_2_ (in liquid) are possible formation pathways [99,100,101]. The production in liquid of H_2_O_2_ and ˙OH by water dissociation/ionization driven by radicals and vacuum UV radiation was shown previously [36,90]. The formation of peroxynitrite by the reaction of HNO_2_ with H_2_O_2_ is favored by low pH (<4), which may be achieved in the gas–liquid interface [99,101] and not in the liquid bulk (Figure S1) [102].

As shown in Figure 4 and Figure 6, the addition of humidity in the working gas reduced the general tyrosine oxidation in conditions containing oxygen, increasing drastically in Ar and Ar/N_2_ the production of nitrotyrosine (up to 2 µM), nitrosotyrosine (one and four −NO groups, 2%), mixed modifications (2%), and multiple nitration events (two −NO_2_ groups, 8%). Conditions with Ar-only in the working gas became slightly more oxidative (25% oxidized tyrosine) than dry conditions. In contrast, tyrosine oxidation was lower than in dry conditions with molecular admixtures (7% and 5% for Ar/O_2_ and Ar/N_2_/O_2_, respectively), as well as the production of tyrosine derivatives (Figure 6).

This can be explained by the interaction of water molecules in the effluent with gaseous species formed using Ar/O_2_ and Ar/N_2_/O_2_ (e.g., ˙O, ^1^O_2_, ˙NO_2_). In parallel, it was shown that this interaction, mostly with Ar_2_^*^ and ˙O, generated ˙OH, ˙H, and ˙O_2_^−^ in the effluent area, which partially diffuse into the target, forming high amounts of H_2_O_2_ by recombination of ˙OH [40,103]. In the gas phase of humidified kINPen plasmas with low O_2_%, a boost of gaseous nitrogen chemistry was also detected, with higher production of HNO_3_ (~4 × 10^13^ cm^−3^) and ˙NO (~6 × 10^13^ cm^−3^), rather than ˙NO_2_, the formation of which depended on O_2_% densities [41].

Considering the 10-times higher production of nitrotyrosine in humid conditions with Ar-only or Ar/N_2_ than in conditions with Ar/O_2_ and Ar/N_2_/O_2_, a higher formation of nitrogen species in liquid is key. Rather than a direct impact of gaseous ˙NO_2_, the formation of peroxynitrite via different pathways is facilitated. The reaction of gaseous or dissolved ˙NO with ˙O_2_^−^ at the interface or in the bulk of the liquid is a substantial pathway. Additionally, the high amounts of HNO_3_ in the gas phase favor an attack of ˙OH, forming ˙NO_3_, which is an unstable species generating H_2_O_2_ and ˙NO_2_ in contact with water. Alternatively, ˙NO_2_ could form HNO_2_ by reacting with water molecules. The subsequent reaction with H_2_O_2_ yielding ONOOH is not favored at the pH of 7.4, precluding this as a major reaction route [102]. At the interface, ˙NO_2_ could form peroxynitrite by the reaction with ˙OH radicals. Table 2 shows an overview of the major modifications induced on tyrosine during plasma treatment, together with the related plasma-derived species in the gas phase and the proposed reactions occurring in liquid to form the attacking species.

### 3.3. Tyrosine Modification Induced by Control Oxidants

**Figure 7:**
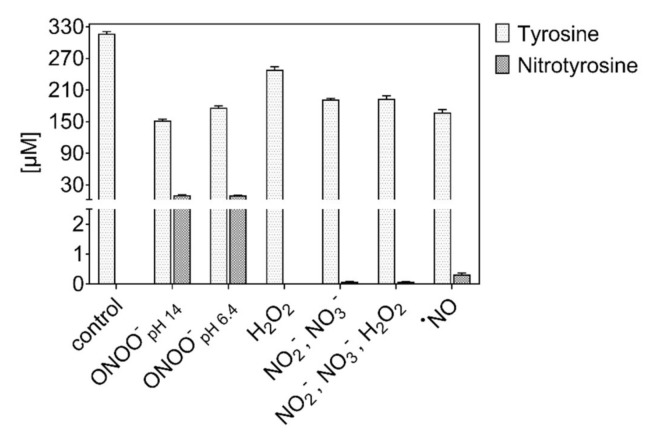
Tyrosine consumption and 3-nitrotyrosine formation by control oxidants. Peroxynitrite efficiently introduced a nitro group, but independently from pH (3 min incubation of 0.3 mM tyrosine in 5 mM ammonium formate, pH 7.4, 300 µM of each control oxidant).

Incubations with control oxidants (NO donor, NO_2_^−^/NO_3_^−^, H_2_O_2_/NO_2_^−^/NO_3_^−^) were performed at neutral pH (7.4). Peroxynitrite was tested at two different pH (pH 14 and 6.4) [83,84]. The observed tyrosine derivatives are shown in Figure 7 (quantitative) and 8 (qualitative). Peroxynitrite was most efficient in modifying tyrosine (46% and 37% at pH 14 and 6.4, respectively). In particular, nitrotyrosine (~10 µM) was formed (Figure 7). The substantial functionalization observed for ONOO^−^ at near neutral pH is due to the protonation of the ion, yielding instable peroxynitrous acid (ONOOH, 58.7% considering pKa = 6.8). This rapidly isomerizes into NO_3_^−^ and H+, and dissociates in ˙OH and ˙NO_2_ (30% yield) [62,66,70]. The incorporation of up to four −NO_2_ groups in the tyrosine structure emphasized the favored formation of ˙NO_2_ by peroxynitrite dissociation. In addition, the single nitration of tyrosine could be induced by the indirect reaction of ONOOH via the formation of a transition intermediate generated in the isomerization of ONOOH to NO_3_^−^ [88]. According to [88,89], the hydroxylation was stronger at lower pH because of the formation of a peroxynitrous acid intermediate that facilitates the cleavage of the ions O-O bond.

**Figure 8:**
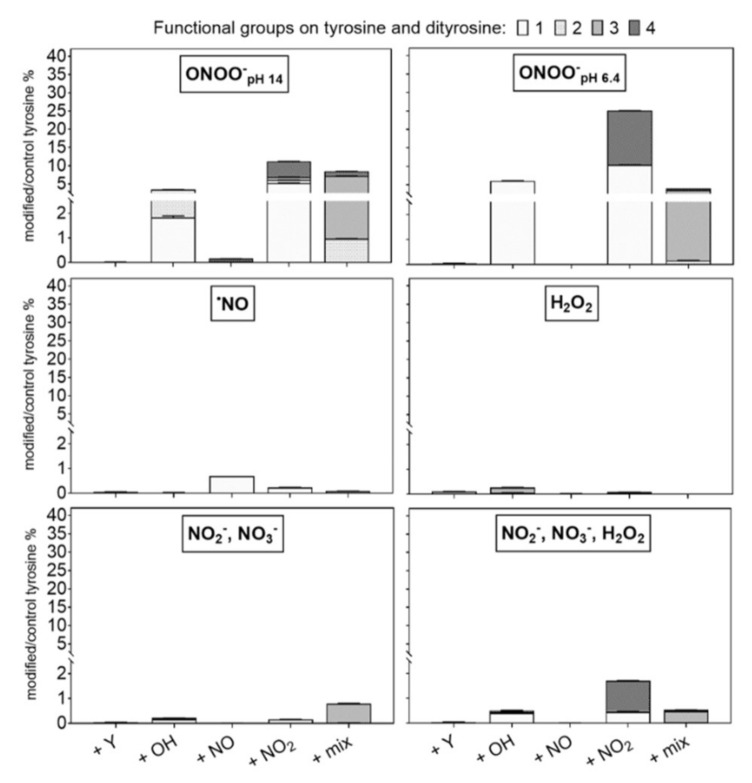
Modifications of tyrosine observed after treatment using control oxidants. Peroxynitrite was most efficient, with higher pH boosting the introduction of multiple groups. Labels: +Y (tyrosyl, +180.066 Da), +OH (hydroxyl, +15.9949 Da), +NO (nitroso, +28.9902 Da), and +NO_2_ (nitro, +44.9851 Da). Up to four groups were observed per molecule (indicated by light to dark grey). The introduction of diverse groups is represented as “mixed” (Compounds 20 to 27, Table 1). Treatment time 3 min, 0.3 mM tyrosine in 5 mM ammonium formate, pH 7.4, direct infusion high-resolution mass spectrometry (MS). Relative compound intensities are given (tyrosine ~4948 counts in control).

However, at pH 14, a similar nitration yield was observed. According to [99], different products generated during ONOO^−^ homolysis (˙NO and ˙O_2_^−^) and decomposition (O_2_ and NO_2_^−^) could be involved in the direct or indirect functionalization of biomolecules, including the peroxynitric anion (O_2_NOO^−^). The functionalization of tyrosine via non-radical processes was confirmed, also inducing a higher incorporation of up to four different functional groups into the tyrosine moiety (+2 −OH groups, two and four mixed groups) (Figure 8). Incubations with any other compound (mix) yielded very low amounts of nitrotyrosine (<0.5 µM), and only the ˙NO donor could induce nitroso-groups on tyrosine, confirming a direct reaction with the aromatic ring (Figure 8) [65]. The low formation of nitrosotyrosine indicates the low reactivity of ˙NO itself with the ring and a limited oxidation of ˙NO to nitrite ions’ respective nitrous acid. A partial oxidation to ˙NO_2_ in the presence of O_2_ could justify the formation of nitrotyrosine.

By the incubation with H_2_O_2_, only 11% of the tyrosine was consumed, and yielded hydroxylated products. When nitrite and nitrate ions were available at the same time, a few nitrations were observed. Of note, no nitrosylation occurred, indicating that peroxynitrite, formed from H_2_O_2_ and NO_2_^−^, was responsible. However, because of the pH of 7.4 in the experiment, this reaction was not favored. Likewise, the pH in the plasma treatment never reached below pH 7, even after 10 min of treatment (Figure S1 in SI), excluding a substantial formation of peroxynitrite in the bulk. When nitrite/nitrate were the sole available compounds, no modification of tyrosine occurs. The apparent consumption of tyrosine in conditions with a high sodium content was overestimated as a result of the formation of tyrosine salts that evaded detection in the applied experimental conditions.

### 3.4. Both Gas Phase- and Liquid Phase-Derived Species Contribute to Tyrosine Modification

**Figure 9:**
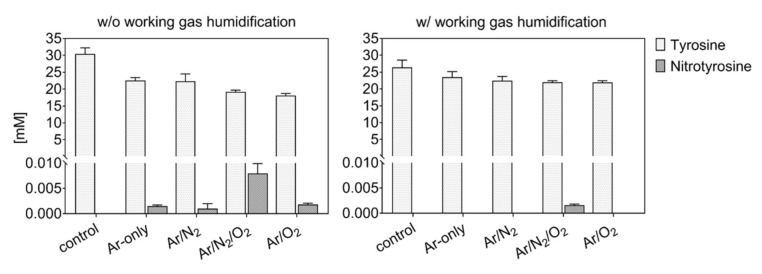
Tyrosine consumption and 3-nitrotyrosine formation by plasma-derived ROS in concentrated tyrosine solutions. The activity of peroxynitrite (humid conditions) is quenched in favor of nitrogen dioxide (dry conditions). Three minutes, 30 mM tyrosine in 5 mM ammonium formate, pH 7.4. Plasma is formed from dry (left) or humid (320 ppm H_2_O, right) working gas.

To investigate the role of solvent-derived reactive species, higher concentrated solutions of tyrosine (30 mM) were treated. The identified products were similar to the more diluted solutions (see Figure S2/dry working gas and Figure S3/humidified working gas for a complete overview). While the total number of oxidized molecules increased, the conversion was proportionally lower and indicated a limitation of the produced ROS/RNS in liquid. In conditions with Ar-only, Ar/N_2_, and Ar/O_2_, a conversion of 3.2 × 10^18^, 3.0 × 10^18^, and 5.0 × 10^18^ molecules per second occurred respectively (Figure 9). This corresponds to a total of 24%, 22%, and 36% tyrosine conversion in derivatives. In comparison, treatments of 0.3 mM tyrosine solutions yielded a conversion rate of 1.8 × 10^16^, 6.6 × 10^16^, and 9.5 × 10^16^ molecules per second, corresponding to 12.8% (Ar-only), 45% (Ar/N_2_), and 66% (Ar/O_2_) converted tyrosine, respectively. On average, the conversion in concentrated tyrosine solutions was a factor of ≈50 fold higher than in diluted solutions, while their concentration was 100-fold higher. This would suggest that, using these working gases and a high tyrosine concentration, the amount of induced modifications was reduced as a result of limited production/action of species in liquid. In parallel, gaseous species were still effective on the target.

In contrast, conditions with both N_2_ and O_2_ in the dry working gas led to an identical proportional formation of nitrotyrosine (0.026% of converted tyrosine) for both concentrations. In this case, the high amounts of generated ˙NO_2_ in the gas phase could directly modify tyrosine molecules at the interface or in the underlying water layers of both high and low concentrated solutions. The apparent reaction probability remained constant, indicating that the interface occupation by the tyrosine molecules did not change with the concentration or that a corresponding decay reaction (e.g., formation of hydroxytyrosine from nitrotyrosine) increased proportionally.

**Figure 10:**
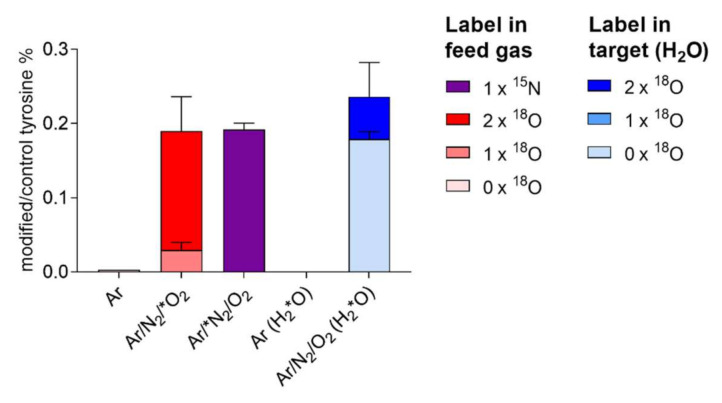
Nitrotyrosine formed from plasma treatment of tyrosine incorporates predominantly gas phase-derived atoms (100% N, ≈80% O). Here, 20% of oxygen atoms are introduced from the solvent (water), indicating a role for water-derived OH radicals (for details, see text). Dry argon working gas contained ^18^O_2_ and ^15^N_2_. In independent experiments, tyrosine was dissolved in labelled water (H_2_^18^O). Measurements performed via direct infusion mass spectrometry in triplicates.

The role of gaseous ˙NO_2_ in forming nitrotyrosine was determined using heavy isotopes (^15^N_2_ or ^18^O_2_, or H_2_^18^O). Almost 80% ^18^O and 100% ^15^N originated from the gas phase (Figure 10), confirming the direct nitration of tyrosine by ˙NO_2_, rather than other species (e.g., peroxynitrite) originating from liquid chemistry.

By introducing humidity in the working gas, the oxidation of tyrosine, as well as the production of nitrotyrosine (Figure 9) and other tyrosine derivatives, such as dityrosine and nitrosotyrosine (Figure S3), were drastically reduced in relation to dry conditions (Figure S2). This suggests that, in humidified conditions, relevant species act and/or are formed in the bulk of the liquid. It was observed that, for working gases with <0.5 O_2_%, the presence of water molecules induced mostly the formation of species such as HNO_3_ and ˙NO, along with water-derived species H_2_O_2_, ˙OH, ˙H, and O_2_^−^˙, rather than highly oxidized gaseous species, such as N_2_O_5_, O_3_, and ˙NO_2_ [41]. When treating 0.3 mM tyrosine solutions with humid plasmas, nitrotyrosine was detected in a 10-fold higher amount than for the respective dry discharges. Considering that high concentrated solutions limited the formation/action of reactive species in liquid, gaseous species formed in humidified working gases (<0.5 O_2_%) acted only in the bulk liquid or most likely as precursors of other nitrating species (e.g., peroxynitrite) formed in liquid.

### 3.5. Identification of Plasma-Derived Reactive Nitrogen Species

In presence of the ˙NO scavenger cPTIO or the peroxynitrite scavenger ergothioneine, the formation of N-containing tyrosine derivatives was differential.

**Figure 11:**
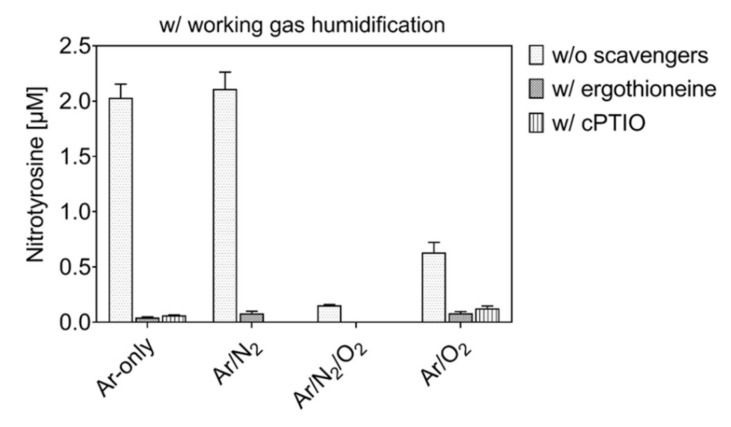
Nitrotyrosine formation by plasma treatment is prevented in the presence of scavengers. Three minute treatment, 0.3 mM tyrosine in 5 mM ammonium formate, pH 7.4, with and without ergothioneine or cPTIO, using Ar ± 1% N_2_/O_2_ as humidified working gas and N_2_ as shielding gas.

A general overview of all identified compounds containing nitrogen is given in Figure S4, while Figure 11 shows quantitative data achieved by quantitative mass spectrometry (HILIC-MRM) for nitrotyrosine. As shown in Figure 11, in conditions with humidified working gases, the production of nitrotyrosine was almost abolished in the presence of cPTIO or ergothioneine. Further, almost all other N-containing tyrosine derivatives were inhibited (Figure S4). These results are in good agreement with the hypothesis that the addition of humidity to the working gas favors the production of peroxynitrite, predominantly originating by the reaction of ˙NO with O_2_^−^˙ derived by water dissociation. The conversion of HNO_3_ in ˙NO_2_ in liquid and its further reaction with ˙OH could still be an additional route for peroxynitrite formation.

The formation and activity of reactive nitrogen species in liquids could not be excluded for dry working gases (Figure S4). Indeed, the formation of some compounds that bear nitroso-groups was prevented only by cPTIO (<0.5% in Ar/N_2_/O_2_, Figure S4a), indicating a role for a direct or indirect role of ˙NO, reformed in the liquid phase from gaseous ˙NO_2_ [81]. Furthermore, almost 2.5% of tyrosine was converted in conditions with dry Ar/N_2_/O_2_ in derivatives scavenged by ergothioneine (Figure S4b,c). These data confirm the formation and chemical activity of peroxynitrite in dry working gas conditions. Finally, conditions with dry working gases produced the maximal amount of nitrogen-containing derivatives in the presence of gas admixtures (3% oxidized tyrosine). Their formation was due paramount to the direct action of gaseous ˙NO_2_ and, to a lesser extent, to a reformation of nitrosative species in the liquid (e.g., H^+^/NO_2_^−^, ONOO^−^). For humidified working gas, an increase of nitrogen-containing derivatives was evident, provided when no oxygen was added (>14% oxidized tyrosine). Here, the nitrosative peroxynitrite is formed in liquid (Figure S4).

However, non-specific reactions must be considered. It was reported that the NO scavenger cPTIO partially reacts with ˙NO_2_ [78], while ergothioneine is able to scavenge ^1^O_2_ and ˙OH [79,104]. No discriminative derivatives produced by the reaction of scavengers with control oxidants could be identified in this work.

### 3.6. Plasma Induces Oxidative and Nitrosative Protein Modifications in Wounds

In order to verify that nitrosative modifications are introduced in vivo as well, the wound proteome of human patients before and after plasma treatment was analyzed. A special emphasis was given to modifications at the tyrosine moiety.

**Figure 12:**
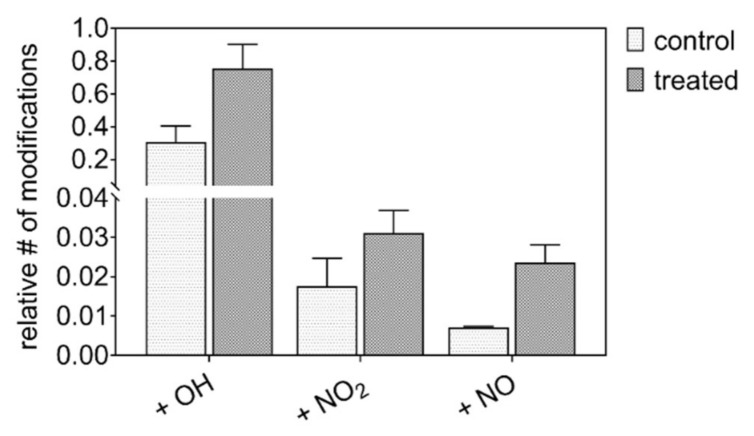
Cold plasma induced post-translational modifications in the proteome of wound exudates of diabetic patients. Proteome analysis performed via nanoLC-MS, with the detection of mass shifts corresponding to the introduced functional group (OH = 15.9949 Da; NO = 28.9902 Da, NO_2_ = 44.9851 Da), according to [80].

Overall, 330 proteins have been identified and were searched for oxidative post-translational modifications (PTMs). PTMs were detected at 80 proteins; 27 of these were modified at tyrosine moieties (Table 3). An increase of 63% (oxidation), 44% (nitration), and 69% (nitrosylation) compared with the control was observed (Figure 12). Target proteins were highly abundant blood plasma and blood cell components (transferrin, albumin, hemoglobin, and so on), or belonged to the wound bed/wound margins (keratins, fibronectin). The modified tyrosine residues were located at the protein surface or otherwise exposed structures. As samples were taken immediately after treatment, regular physiologic reactions were unlikely and the increase of modifications was attributed directly to the impact of plasma-derived reactive species. The oxidation of tyrosine and cysteine residues in blood plasma proteins was already observed for inflammatory levels of hypochlorous acid [105]. Target proteins were, among others, complement C3 and apolipoprotein A-I, and albumin was affected in this study as well. Cold plasma-derived atomic oxygen forms hypochlorite anions upon reaction with chloride ions locally, which subsequently serves as oxidants yielding di-tyrosine, hydroxylations, and chlorinations (rare). The introduction of nitro and nitroso groups into tyrosine occurs by local formation of peroxynitrite and NO_2_ radicals, which are only effective at or close to the treated surface. Yielding a higher reactivity of the tyrosine’s phenolic hydroxyl group, significant effects on the regulation of cell migration, angiogenesis, and mast cell degranulation [67,72] were described for physiologic conditions (nitric oxide pathway). The proportional strong oxidation/nitration of haptoglobin, a protein protective against hemoglobin related oxidative stress, and of bulky structural proteins like fibronectin and keratins, confirms a significant presence of reactive species from the plasma discharge and the proteins’ roles as scavenger molecules.

The introduction of nitro groups into proteins by plasma-derived species suggests at least a contributing role in the observed crosstalk to cellular redox signaling pathways involved in acute and chronic wound healing, well in line with a recent study that emphasized the role of target cell stimulation over anti-microbial effects [22]. The results of this study suggest that the clinical effectivity of cold plasmas would benefit from a switch from the current condition (argon only) to argon/N_2_/O_2_ as a working gas to increase the impact of the plasma-derived RNS on biomolecules and subsequent signaling events.

## 4. Summary and Conclusions

This work studied the liquid chemistry of argon plasmas generated by the kINPen, with a special focus on reactive nitrogen species. Assuming that the liquid chemistry is the bridge between the gaseous plasma and biological systems, we looked for the impact of nitrogen species on the model biomolecule tyrosine. Using a mass spectrometry driven approach, 26 different compounds were identified. Their respective pattern was exploited to determine the dominant reactive species formed in the gas or in/at the liquid phase.

Nitration reactions were significant for dry Ar/O_2_/N_2_ plasma. Gaseous ˙NO_2_ was found to be responsible for a direct nitration of tyrosine at the gas–liquid interface. Additionally, it is involved in the formation of peroxynitrite and nitric oxide radicals. While the relevance of nitric oxide for the product formation was limited, peroxynitrite contributed to a substantial extent. When water molecules were present in the working gas, its role was further emphasized owing to a higher peroxynitrite formation via additional pathway; that is, the reaction of gaseous nitric oxide with superoxide anion radicals and the reaction of hydrogen peroxide and nitrous acid/nitrite at the gas–liquid interface. In conditions with a high prevalence of ROS (e.g., dry Ar/O_2_), the impact of RNS was minimized. Here, stable species like N_2_O_5_ that do not penetrate the interface evolve from ˙NO_2_ in the gas phase and the formation of peroxynitrite decreases. Because of the high activity of ROS at the interface and the liquid bulk, potentially formed nitrated/nitrosylated tyrosine products are eliminated in favor of hydroxylated compounds. Finally, the introduction of nitroso and nitro groups into proteins in vivo by cold plasma treatments was confirmed in human diabetes 2-related chronic wounds. The tyrosine moiety was particularly attacked, allowing for changes in the protein functionality. This suggests a contribution of plasma-derived RNS via covalent changes to the observed efficacy in wound healing.

**Figure 13:**
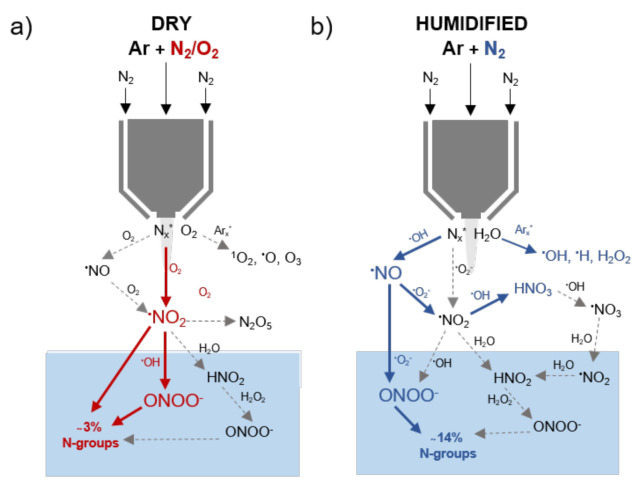
Suggested chemical pathways for the production of bioactive RNS species in plasma treated tyrosine solutions in dry (**a**) and humidified (**b**) conditions. Represented species were previously detected for the kINPen [41,82,100], except for peroxynitrite. Pathway confirmed in liquids (compact lines) by use of scavengers or isotope labels.

In conclusion, this work verified the controllability of kINPen plasmas to achieve a relevant production of reactive nitrogen species (Figure 13). The dissolved or at the interface generated species, especially ˙NO_2_ and ONOO^−^, led to nitrosative reactions on biomolecules, in both complex and model conditions. A relevant contribution to the observed biomedical effects of plasmas must be assumed.

## Figures and Tables

**Table 1:** Overview of modifications introduced in the tyrosine (Y) structure by tuning kINPen cold plasmas. * Assignment of a group letter in relation to the results obtained from the experiment performed in the presence of scavengers (details in Paragraph 3.5, Figure S4). The areas of compounds scavenged in the same way were summed up to generate six groups (from a to f).

Functional Group(s) on Tyrosine	Formula	[M+H]^+^	Compound Code	Group Letter *
None	C_9_H_11_NO_3_	182.081725	1	-
1 × OH	C_9_H_11_NO_4_	198.0766	2	-
2 × OH	C_9_H_11_NO_5_	214.071525	3	-
3 × OH	C_9_H_11_NO_6_	230.066425	4	-
4 × OH	C_9_H_11_NO_7_	246.061425	5	-
1 × NO	C_9_H_10_N_2_O_4_	211.071925	6	e
2 × NO	C_9_H_9_N_3_O_5_	240.062025	7	a, d
3 × NO	C_9_H_8_N_4_O_6_	269.052225	8	b, d
4 × NO	C_9_H_7_N_5_O_7_	298.042325	9	b, d
1 × NO_2_ + 1 × OH, 1 × NO	C_9_H_10_N_2_O_5_	227.066825	10	c, e
2 × NO_2_ + 2 × OH, 2 × NO	C_9_H_9_N_3_O_7_	272.051825	11	b, e
3 × NO_2_	C_9_H_8_N_4_O_9_	317.036925	12	b, e
4 × NO_2_	C_9_H_7_N_5_O_11_	362.022025	13	b, e
1 × Y	C_18_H_20_N_2_O_6_	361.139925	14	-
1 × Y, 1 × OH	C_18_H_20_N_2_O_7_	377.134925	15	-
1 × Y, 2 × OH	C_18_H_20_N_2_O_8_	393.129825	16	-
1 × Y, 3 × OH	C_18_H_20_N_2_O_9_	409.124725	17	-
1 × Y, 1 × NO	C_18_H_19_N_3_O_7_	390.130125	18	c, d
1 × Y, 1 × NO_2_ + 1 × Y, 1 × OH, 1 × NO	C_18_H_19_N_3_O_8_	406.125025	19	e
1 × OH, 2 × NO	C_9_H_9_N_3_O_6_	256.056925	20	a, f
1 × OH, 3 × NO	C_9_H_8_N_4_O_7_	285.047125	21	b, f
1 × OH, 1 × NO_2_ + 2 × OH, 1 × NO	C_9_H_10_N_2_O_6_	243.061725	22	c, f
2 × OH, 1 × NO_2_ + 3 × OH, 1 × NO	C_9_H_10_N_2_O_7_	259.056625	23	f
3 × OH, 1 × NO_2_	C_9_H_10_N_2_O_8_	275.051525	24	f
1 × OH, 2 × NO_2_	C_9_H_9_N_3_O_8_	288.046825	25	f
2 × OH, 2 × NO_2_	C_9_H_9_N_3_O_9_	304.041725	26	a, f
1 × OH, 3 × NO_2_	C_9_H_8_N_4_O_10_	333.031825	27	f

**Table 2:** Overview of the most relevant tyrosine derivatives observed in solution, potentially related plasma-derived species [41,81,97], and proposed reactions occurring in the liquid [15,81,99,100,101].

	Added Functional Groups on Tyrosine	Plasma Components in the Gas Phase	Proposed Reactions in Liquid
**Ar-only**	1 × OH	Ar_2_^*^, VUV	H_2_O → ˙H + ˙OH
**Ar + N_2_**	1–2 × OH	Ar_2_^*^, VUV	H_2_O + *hv* → ˙H + ˙OH
**Ar + N_2_ + O_2_**	1–2 × OH; 1, 4 × NO; 1–2 × NO_2_	˙NO_2(g)_, ˙O_(g)_	˙O + H_2_O → ˙OH + ˙OH ˙NO_2_ + ˙OH → ONOOH
**Ar + O_2_**	1–4 × OH; 1, 4 × NO; 1–2 × NO_2_; mi×ed	˙NO_2(g)_, ˙O_(g)_, O_3(g)_ N_2_O_5(g)_	˙O + H_2_O → ˙OH + ˙OH ˙NO_2_ + ˙OH → ONOOH
**Ar-only hum**	1 × OH; 1, 4 × NO; 1–2 × NO_2_; mi×ed	˙NO_(g)_, HNO_3(g)_, H_2_O_2 (aq)_, ˙O_2_^−^_(aq)_, ˙OH _(aq)_	˙NO + ˙O_2_^−^ → ONOOH HNO_2_ + H_2_O_2_ → ONOOH
**Ar + N_2_ hum**	1 × OH; 1, 4 × NO; 1–2 × NO_2_; mi×ed	˙NO_(g)_, HNO_3(g)_, H_2_O_2 (aq)_, ˙O_2_^−^_(aq)_, ˙OH _(aq)_	˙NO + ˙O_2_^−^ → ONOOH HNO_2_ + H_2_O_2_ → ONOOH
**Ar + N_2_ + O_2_ hum**	1 × OH; 1 × NO; 1 × NO_2_	˙NO_2(g)_, N_2_O_5(g)_, H_2_O_2 (aq)_, ˙O_2_^−^_(aq)_, ˙OH _(aq)_	˙NO_2_ + ˙OH → ONOOH HNO_2_ + H_2_O_2_ → ONOOH
**Ar + O_2_ hum**	1 × OH; 1 × NO; 1 × NO_2_	˙NO_2(g)_, N_2_O_5(g)_, H_2_O_2 (aq)_, ˙O_2_^−^_(aq)_, ˙OH _(aq)_	˙NO_2_ + ˙OH → ONOOH HNO_2_ + H_2_O_2_ → ONOOH

**Table 3:** Twenty seven proteins were identified from wound exudates carrying oxidative modifications on tyrosine (oxidation/+15.99 Da, nitrosylation/+28.99 Da, nitration/+44.99 Da). The total number of proteins found with modification was 80 out of 308 proteins identified. Blood-derived proteins dominate the list. Modifications occur predominantly at the protein surface.

Uniprot Identifier	Abbreviated Protein Name	Protein Name	Modified Tyrosine Residue (Bold Denotes Significant Site)
**P02768**	ALBU	Albumin	**Y54**, Y172, Y174, **Y287**, **Y377**, Y425, Y356, **Y365**, Y521
**P01009**	A1AT	Alpha-1-antitrypsin	Y184
**P01023**	A2MG	Alpha-2-macroglobulin	Y544, Y818, Y1152, Y1264
**O43299**	AP5Z1	AP-5 complex subunit zeta-1	**Y344**
**P02647**	APOA1	Apolipoprotein A-I	Y124, Y190
**P00915**	CAH1	Carbonic anhydrase 1	Y21
**P00450**	CERU	Ceruloplasmin	Y55, Y539
**P01024**	CO3	Complement C3	Y139, Y1447, Y1620
**P0C0L4**	CO4A	Complement C4-A	Y1612
**P02751**	FINC	Fibronectin	Y841, Y2362
**P00738**	HPT	Haptoglobin	Y224, **Y242**, **Y280**, Y386, Y389
**P69905**	HBA	Hemoglobin subunit alpha	**Y25, Y43**
**P68871**	HBB	Hemoglobin subunit beta	**Y36, Y131**
**P02042**	HBD	Hemoglobin subunit delta	Y36, Y131
**P01876**	IGHA1	Immunoglobulin heavy constant alpha 1	Y276
**P01857**	IGHG1	Immunoglobulin heavy constant gamma 1	**Y161**, Y319
**Q14624**	ITIH4	Inter-alpha-trypsin inhibitor heavy chain H4	Y157
**P13645**	K1C10	Keratin, type I cytoskeletal 10	Y172, Y325
**P02533**	K1C14	Keratin, type I cytoskeletal 14	Y46
**P35527**	K1C9	Keratin, type I cytoskeletal 9	Y330, Y345
**P04264**	K2C1	Keratin, type II cytoskeletal 1	Y266, Y295, Y358, Y373
**Q7Z794**	K2C1B	Keratin, type II cytoskeletal 1b	Y361
**P35908**	K22E	Keratin, type II cytoskeletal 2	Y563, Y589
**P02788**	TRFL	Lactotransferrin	**Y211, Y545**
**P32119**	PRDX2	Peroxiredoxin-2	Y126
**P00747**	PLMN	Plasminogen	Y66
**P02787**	TRFE	Serotransferrin	Y114, Y333, Y533

## Supplementary Materials

The following are available online at https://www.mdpi.com/2218-273X/10/12/1687/s1, Figure S1: pH measurements performed after 30, 180 and 600 seconds plasma treatment of 0.3 mM tyrosine solutions (in 5 mM ammonium formate) using different working gas admixtures (Ar ± 1% N2/O2); Figure S2: Modifications of tyrosine observed after plasma treatment using dry working gas of high concentrated tyrosine solutions; Figure S3: Modifications of tyrosine observed after plasma treatment using dry working gas of high concentrated tyrosine solutions; Figure S4: Modifications of tyrosine observed after plasma treatment using dry and humidified working gases of normal tyrosine solutions and in presence of scavengers.
