# Supplementary material for: On the Liquid Chemistry of the Reactive Nitrogen Species Peroxynitrite and Nitrogen Dioxide Generated by Physical Plasmas
Source: Biomolecules. 2020 Dec 16;10(12):1687. doi: 10.3390/biom10121687 (PMC7766045; doi:10.3390/biom10121687)
Supplement: Supplementary file 1 [file biomolecules-10-01687-s001.pdf]

On the liquid chemistry of the reactive nitrogen species peroxynitrite and nitrogen dioxide generated by physical plasmas

G. Bruno[1], S. Wenske[1], J.-W. Lackmann[2], M. Lalk[3], T. von Woedtke[4], K. Wende[1*]

[1]*ZIK plasmatis, Leibniz Institute for Plasma Science and Technology, Greifswald, Germany*

[2]*Cluster of Excellence Cellular Stress Responses in Aging-Associated Diseases, University of Cologne, Cologne, Germany*

[3]*Institute of Biochemistry, University of Greifswald, Greifswald, Germany*

[4]*Leibniz Institute for Plasma Science and Technology, Greifswald, Germany*

Keywords: cold physical plasmas, kINPen, redox signaling, reactive nitrogen species, nitrosative stress, PTMs

*Corresponding author (email: kristian.wende@inp-greifswald.de)

SUPPLEMENTARY MATERIAL

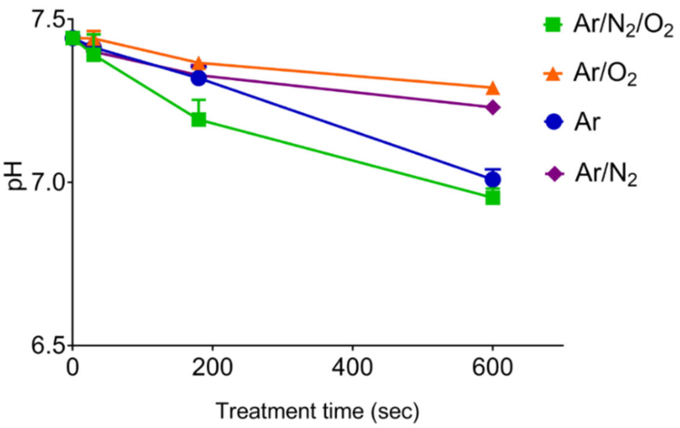

Figure S1. pH measurements performed after 30, 180 and 600 seconds plasma treatment of 0.3 mM tyrosine solutions (in 5 mM ammonium formate) using different working gas admixtures (Ar ± 1% N_2/O_2).

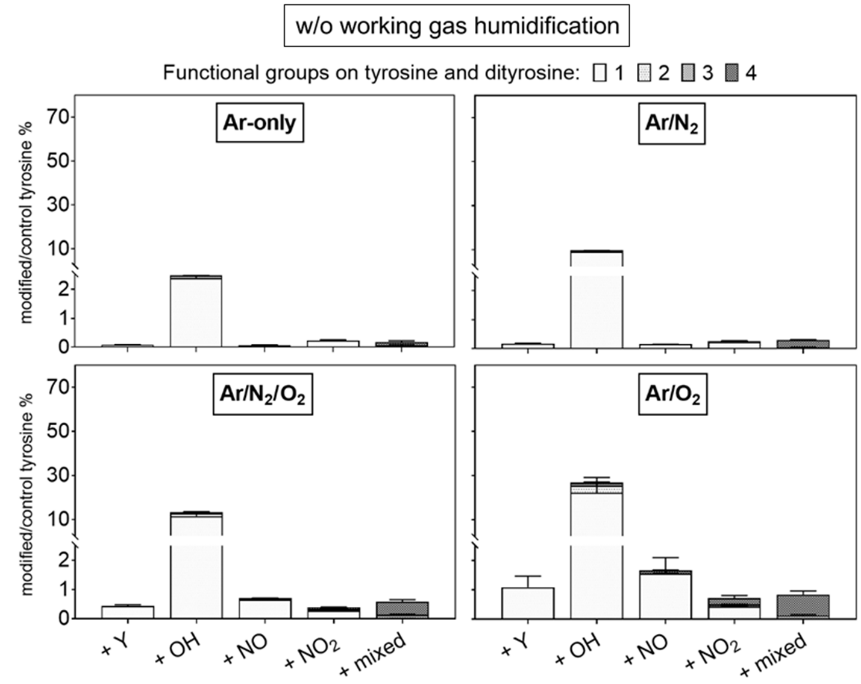

Figure S2. Modifications of tyrosine observed after plasma treatment using dry working gas of high concentrated tyrosine solutions. Labels: + Y (tyrosyl, +180.066 Da), + OH (hydroxyl, +15.9949 Da), + NO (nitroso, +28.9902 Da), and + NO_2 (nitro, +44.9851 Da). Up to four groups were observed per molecule (indicated by light to dark grey). The introduction of diverse groups is represented as “mixed” (Compounds 20 to 27, Table 1). Treatment time 3 min, 30 mM tyrosine in 5 mM ammonium formate, pH 7.4. Tyrosine solutions diluted 1:100 after treatment and before analysis. Relative compound intensities are given (tyrosine ~ 4321 counts in control).

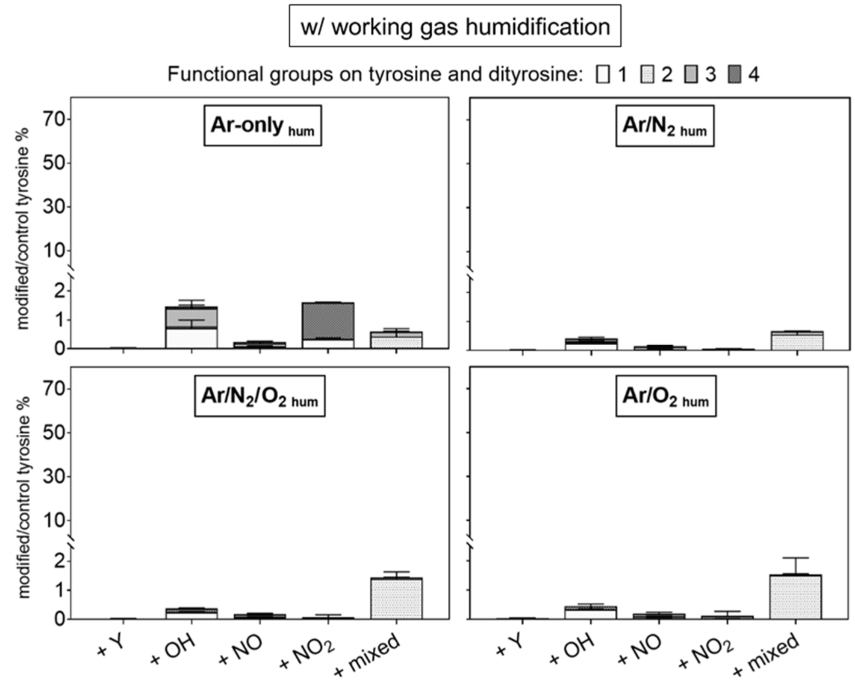

Figure S3. Modifications of tyrosine observed after plasma treatment using dry working gas of high concentrated tyrosine solutions. Labels: + Y (tyrosyl, +180.066 Da), + OH (hydroxyl, +15.9949 Da), + NO (nitroso, +28.9902 Da), and + NO_2 (nitro, +44.9851 Da). Up to four groups were observed per molecule (indicated by light to dark grey). The introduction of diverse groups is represented as “mixed” (Compounds 20 to 27, Table 1). Treatment time 3 min, 30 mM tyrosine in 5 mM ammonium formate, pH 7.4. Tyrosine solutions diluted 1:100 after treatment and before analysis. Relative compound intensities are given (tyrosine ~ 4271 counts in control).

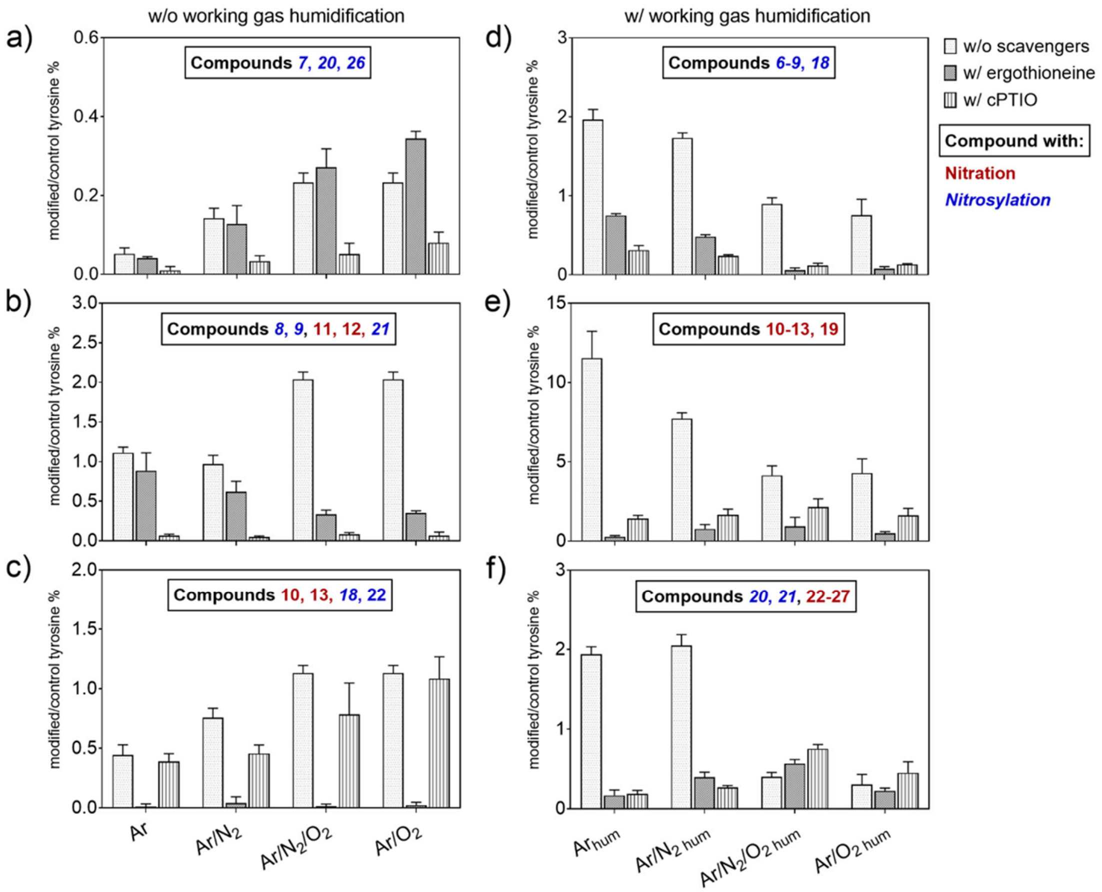

Figure S4. Modifications of tyrosine observed after plasma treatment using dry and humidified working gases of normal tyrosine solutions and in presence of scavengers. Labels: + Y (tyrosyl, +180.066 Da), + OH (hydroxyl, +15.9949 Da), + NO (nitroso, +28.9902 Da), and + NO_2 (nitro, +44.9851 Da). Up to four groups were observed per molecule (indicated by light to dark grey). The introduction of diverse groups is represented as "mixed" (Compounds 20 to 27, Table 1). . Compounds (code indicated in Table 1) values were summed up for groups showing the same trends. The presence of nitro (in red) or nitroso (cursive, in blue) groups in the structures is highlighted. Treatment time 3 min, 30 mM tyrosine in 5 mM ammonium formate, pH 7.4. Relative compound intensities are given (tyrosine ~ 4271 counts in control).
